# Dr. Horace M. Paine

**Published:** 1904-01

**Authors:** 


### OBITUARY.

Dr. Horace M. Paine, formerly of Albany, died at the home of his son at Atlanta, Ga., December 6, 1903, aged 76 years. He graduated from the medical department of the University of the City of New York in 1849, and became one of the most respected homeopathic physicians in this country. Dr. Paine was an early and ardent advocate of state examination for license and did much to bring about this reform. His remains were taken to Albany for burial.

